# PP50 Improving The Evaluation Of Devices And Procedures: Impact Of The National Committee For Health Technology Incorporation Subcommittee

**DOI:** 10.1017/S0266462325102699

**Published:** 2025-12-29

**Authors:** Aíla Carmo, Barbara Pozzi Ottavio, Daniele Marques, Joana Silva, Luciana Xavier, Luciene Bonan, Priscila Louly

## Abstract

**Introduction:**

From the 1,255 technologies submitted to Brazil’s National Committee For Health Technology Incorporation (CONITEC) from its creation in 2012 to November 2024, 26 percent were either products or procedures. The specifics of these technologies, from the available evidence to their implementation, have consequences for the health technology assessment (HTA) process. To deal with these, CONITEC was divided into three committees, one of which specializes in health products and procedures, along with a subcommittee to support it.

**Methods:**

The aim was to present how the Products and Procedures Subcommittee has contributed to improving CONITEC’s evaluations since its first meeting in May 2023. A qualitative evaluation was conducted using the Subcommittee’s meeting minutes and CONITEC’s recommendation reports to identify technologies reviewed by the Subcommittee and its inputs into the HTA reports. The name of the technology and its indication, evaluation status, and theme were extracted from CONITEC’s website for each of the products or procedures submitted for evaluation.

**Results:**

The subcommittee evaluated 29 topics, including three products and 19 procedures, that were assessed by CONITEC. The remaining seven were conformity analyses, whereby documents are assessed for completeness and the eligibility of the technology for the HTA process is evaluated with respect to the health system’s norms. The most frequent topic areas were oncology (38%), neurology (10.3%) and gastroenterology (10.3%). The subcommittee supported CONITEC by providing data or discussing, for instance the installed capacity of positron emission tomography and computed tomography; potential barriers to implementing cervical cancer screening; the technical specificities of urinary porphobilinogen assay tests; and expanding nomenclature to enable competition on the Rapid Direct Multiplex Polymerase Chain Reaction Test.

**Conclusions:**

The subcommittee has improved the HTA process by bringing together stakeholders from within and outside the Ministry of Health. It enables a broader evaluation of technologies and facilitates technical improvements, corrections, and the incorporation of additional data. This streamlines the HTA process and technology implementation by anticipating challenges and fostering collaboration to articulate effective solutions.

